# Kinetic study and modelling of drying of *Chlorellavulgaris*

**DOI:** 10.1016/j.heliyon.2024.e38441

**Published:** 2024-09-26

**Authors:** Debabrata Karmakar, Nishat Tasnim, Md. Rakibul Hasan, Md. Saddam Hossain, Paroma Arefin, Dip Bhowmik, Yead Morshed Nibir, Kazi Bayezid Kabir, Rezaul Karim

**Affiliations:** a Bangladesh Council of Scientific and Industrial Research (BCSIR), Dhaka, Bangladesh; b Department of Chemical Engineering, Bangladesh University of Engineering and Technology, Dhaka, Bangladesh

**Keywords:** Microalgae, Thermogravimetric analysis, Differential scanning calorimetry, Drying kinetics, Semi-theoretical models

## Abstract

Microalgae, a versatile source of biofuels, chemicals, and nutraceuticals, necessitates efficient drying for subsequent applications. Extensive studies have been done on the benefits and uses of microalgae, but very few are focusing on drying. This research focused on a specific microalga, *Chlorella vulgaris*, to analyze the drying kinetics involved in the moisture removal process. Data on drying behavior were collected using thermogravimetric analysis (TGA) and differential scanning calorimetry (DSC). As the temperature rose, the moisture content of the biomass rapidly decreased and peaked between 65 and 80 °C. From four widely used drying kinetics models, which are typically used to analyze the drying kinetics of agricultural goods, four non-isothermal drying models were derived. These models were assessed using the coefficient of determination (R^2^) and reduced chi-square (χ^2^). Page's model emerged as the best fit for describing drying kinetics. This study introduces a novel approach to characterize the intrinsic properties of freshly harvested *Chlorella vulgaris* by employing TGA and DSC. Unlike other studies focusing on conventional drying methods, our investigation provides real-time insights into the microalgae's thermal behavior during drying.

## 1 Introduction

“Algae” is a colloquial term and refers to a large group of photosynthetic eukaryotes. It can grow almost anywhere and hence plays an important role in the ecosystem. They participate in the bio-remediation process by removing heavy metals from water bodies, as a feedstock in health-supplements, animal feeds, bio-char, bio-sorbents, or for the production of bio-energy [1]. Algal biomass offers a number of advantages over conventional biomass due to high productivity, use of non-productive land, recovery of waste-nutrients, effective sequestration of greenhouse gas, etc. [2]. Recently, microalgae have become more favored than macroalgae due to their superior growth rates, increased yields, and higher lipid content [3]. Among the varieties of microalgae, *Chlorella vulgaris*, which can be easily cultured, display the best suitability toward large scale oil production [4]. Generally, freshly harvested microalgae contain more than 80%–90 % of moisture, which needs to be reduced to about 10 % for any useful application [5]. To optimize oil extraction from algae, the moisture content must be reduced to around 10 % [6]. Therefore, drying is the most important part for making a viable product from microalgae.

Several studies have explored thin layer drying kinetics for various matrices. These studies typically involve experimental drying under controlled conditions, followed by the application of mathematical models to describe the drying behavior. Togrul and Pehlivan (2002) investigated the drying kinetics of apricots and found that the logarithmic model best described the drying behavior [7]. Likewise, Doymaz (2004) investigated the drying kinetics of okra and found that the Page model offered the most accurate representation of the experimental data [8]. Research on the thin layer drying of grains such as corn, wheat, and rice has been extensive. For example, Park et al. (2018) studied the drying characteristics of soybean (*glycine max*) using continuous drying and intermittent drying and concluded that the Midilli-Kucuk model accurately represented the drying process [9]. Similarly, the drying behavior of various herbs and spices, including basil and mint, has been modeled to optimize drying conditions. Doymaz (2006) discovered that the thin layer drying kinetics of mint leaves were well represented by the logarithmic model [10]. Chen et al. (2020) examined the drying characteristics of ammonium polyvanadate subjected to microwave heating, employing a thin-layer drying kinetics fitting model, highlighting the unique behavior of chemical compounds during microwave drying [11]. Similarly, Ding et al. (2021) analyzed the dynamic characteristics and microstructural changes of Sc_2_O_3_-ZrO_2_ ceramic powders during microwave drying, emphasizing the importance of understanding microstructural changes [12].

But drying kinetics of microalgae have not been studied extensively yet. Some studies have explored various drying methods for different microalgae species. Behera and Balasubramanian (2021) investigated the convective and microwave drying kinetics of *Chlorella vulgaris*, developing models to describe the drying behavior under different conditions [13]. Similarly, Schmid et al. (2022) assessed the quality of biomass dried using an industrial solar dryer compared to conventional freeze drying for *Tetraselmis chui* and *Nannochloropsis oceanica*, demonstrating the potential for solar drying as a sustainable alternative [14]. Vilatte et al. (2023) examined the viability of spray drying for preserving recombinant proteins in *Chlamydomonas reinhardtii*, highlighting the importance of optimizing drying parameters to maintain protein integrity [15]. Furthermore, Hosseinizand et al. (2018) investigated *Chlorella vulgaris*'s drying mechanism and identified the optimal drying temperature to maintain its qualitative attributes. They discovered that both low and high drying temperatures negatively impacted the surface color, structure, and composition of carbohydrates and lipids in the microalgae, indicating that an optimal drying temperature range of 60–80 °C is recommended [5] which aligns with the results found in this study.

In this study freshly harvested microalgae paste has been used for TGA analysis to capture the most accurate representation of the sample's thermal behavior without the potential alterations induced by drying. While this approach presents challenges due to the high moisture content, we addressed this by determining moisture content separately. This allowed us to obtain valuable insights into the thermal properties of the fresh microalgae, providing a unique perspective on its behavior. TGA can provide real-time information on the evolution of the sample as it dries, offering insights into the kinetics of water loss and potential changes in the organic matrix. It also helps the detection of volatile compounds as fresh samples may contain volatile compounds that are lost during drying, which TGA can help identify. TGA also can monitor the initial stages of post-harvest degradation, helping to identify critical factors affecting product quality. There was a lot of challenge to perform this. Rapid analyses were performed (quickly as possible after harvesting) to minimize post-harvest changes. A small sample size was used to reduce the impact of water on the instrument. For TGA analysis fixed heating rate has been used in this study.

With a fixed heating rate, TGA provides a consistent and controlled environment, enabling precise measurement of weight loss at a constant temperature increase. This uniformity allows for straightforward analysis and comparison of drying kinetics, ensuring reproducibility of results. To obtain a more comprehensive understanding of drying kinetics, combining both fixed and varying heating rate methods can be beneficial. By leveraging both fixed and varying heating rates, TGA facilitates a comprehensive analysis, enhancing the accuracy and applicability of drying kinetic studies across different materials and industrial scenarios.

The present study contributes significantly to the current research area by providing a detailed and precise analysis of the drying kinetics of *Chlorella vulgaris* using thermogravimetric analysis (TGA). This study enhances the understanding of how different heating rates impact the drying process, offering a comparative perspective that highlights the advantages of using TGA over traditional methods like solar and microwave drying to know the drying kinetics accurately. By using TGA, the study offers highly accurate and controlled data on the drying kinetics of *Chlorella vulgaris*, facilitating a deeper understanding of the thermal stability and decomposition patterns of the microalgae. This precision is particularly beneficial for optimizing industrial drying processes. It also provides a comparative analysis of two fixed heating rates, revealing critical insights into how different drying conditions affect kinetics. This information is valuable for designing more efficient and effective drying protocols tailored to specific industrial needs.

Through the detailed examination of thermal decomposition and phase transitions, the study helps identify optimal drying conditions that preserve the quality and bioactive compounds of *Chlorella vulgaris*. This is crucial for applications in the food, pharmaceutical, and biofuel industries where product quality is paramount. The findings of the study have practical implications for improving drying processes in various industries.

In summary, this study not only advances the scientific understanding of microalgae drying kinetics but also provides practical guidelines for optimizing industrial drying processes, thereby contributing to both academic research and industry practices.

### 1.1 Drying methods

In the context of drying *Chlorella vulgaris* microalgae post-harvest, various drying methods are explored. These techniques aim to reduce moisture content, curtail deterioration, and limit microbial activity. When examining various drying methods for microalgae, including open sun and solar drying, hot air oven drying, microwave drying, spray drying, and freeze drying, each technique presents distinct impacts on the quality of the dried product. Here's a comprehensive analysis of these methods:

Hot air oven drying provides controlled and uniform heating, leading to a consistent and predictable drying process. It allows for better control over drying parameters like temperature and airflow. However, extended exposure to high temperatures can cause thermal degradation of heat-sensitive compounds, including essential fatty acids, pigments, and vitamins. This method can also lead to changes in the physical structure of microalgae, potentially affecting their functional properties [16].

Microwave drying is a rapid and efficient method that significantly reduces drying time. It provides uniform heating, helping to preserve the nutritional and bioactive components of microalgae. Despite its advantages, uneven heating and the formation of hotspots can occur, potentially leading to localized thermal degradation. Additionally, the equipment cost is relatively high compared to more conventional methods, requiring careful optimization to prevent overheating and degradation [17].

Spray drying is a highly efficient method that produces fine, consistent powders with a controlled particle size. It is particularly suitable for producing microalgae powders that are easy to handle and have a long shelf life. The rapid drying process minimizes thermal exposure, preserving most bioactive compounds. However, the initial setup and operational costs are relatively high. The process entails atomizing the microalgae suspension into small droplets, which may occasionally result in the loss of volatile compounds. Additionally, spray drying requires pre-processing steps such as homogenization, which can affect overall process efficiency [18].

Freeze drying preserves the highest amount of nutritional and bioactive compounds because it operates at low temperatures, minimizing thermal degradation. It maintains the physical structure, color, and texture of the microalgae, resulting in a high-quality product. Freeze drying also offers the longest shelf life due to the low residual moisture content. However, this method involves high energy consumption and operational costs, requires specialized equipment, and has a longer drying time compared to other methods. The process is more complex and requires careful optimization [16].

All these different methods of drying have different impacts on the quality of dried products. Freeze drying and spray drying are the most effective in preserving proteins, essential fatty acids, vitamins, and pigments due to their low thermal impact and rapid processing. Microwave drying also preserves nutritional content relatively well but requires careful control to avoid hotspots. Hot air oven drying, and open sun and solar drying tend to cause more degradation of these sensitive nutrients.

In case of preserving bioactive compounds, freeze drying is superior in retaining bioactive compounds such as antioxidants and anti-inflammatory agents, followed by spray drying and microwave drying. Hot air oven drying, and open sun and solar drying are less effective due to prolonged heat and light exposure [19].

Freeze drying excels in preserving microalgae's physical attributes, resulting in products with superior texture, color, and structure. Spray drying produces fine powders but might compromise physical integrity under certain conditions. Microwave, oven, and sun drying often lead to changes in texture and color, with the latter two methods also contributing to lower product quality due to potential contamination. Freeze drying and spray drying also extend product shelf life by effectively reducing moisture content, while the other methods may result in shorter shelf life due to higher residual moisture [19,20].

In summary, the choice of drying method significantly impacts the quality of dried microalgae, with each method having its own set of advantages and drawbacks. Freeze drying and spray drying are generally superior in preserving nutritional and bioactive components and maintaining the physical properties of microalgae, whereas open sun and hot air oven drying are more economical but can compromise the quality of the final product. Microwave drying offers a good balance of efficiency and quality preservation when carefully controlled. Ultimately, the choice of an optimal drying approach hinges on specific product requisites and the paramount importance of maintaining quality throughout the process [21]. Thermogravimetric analysis (TGA) can be instrumental in understanding and optimizing these drying processes to achieve the desired quality outcomes.

### 1.2 Mechanism of drying

Drying refers to the process of eliminating water or other liquids from various materials. The drying mechanisms typically involve surface diffusion from the pore surfaces, capillary action, and diffusion driven by moisture gradients [22]. Drying involves transferring mass as water vapor from the solid to the surroundings, while heat moves from the surroundings to the product, and the drying rate hinges on both these heat and mass transfer processes. The typical drying pattern for hygroscopic materials entails a constant rate period followed by a falling rate period, ceasing once equilibrium moisture content is reached. When the material reaches the crucial moisture content, the material's outer layer initially appears to dry out and the initial falling rate period starts. Liquid diffusion predominantly influences this period, driven by concentration disparities and internal factors like moisture content, temperature, and material structure. The first falling rate period transitions into the second falling rate period, where vapor diffusion becomes crucial, driven by differences in moisture concentration and the internal conditions of the material.

### 1.3 Thin layer drying process

Drying process almost always has a temperature gradient between the air and the temperature of the material to be dried. There is also a temperature gradient along the depth of the solid mass. But by reducing thickness of the materials can easily minimize these gradients to an acceptable value. The thin layer concept pertains to a layer of material with a sufficiently slim thickness to maintain uniform air characteristics throughout, and it involves drying materials fully exposed to drying air. Drying processes are typically categorized into two stages: the constant drying rate phase and the falling drying rate phase. In the former, materials with high moisture content behave somewhat like a liquid surface, akin to an open water body, and drying rate depends on environmental conditions and moisture content. Most drying for solids, particularly agricultural products, occurs during the latter phase, which is constrained by the material's equilibrium moisture content. This falling rate period can be further divided into first and second falling rate periods [23].

#### 1.3.1 Theory of drying kinetics studies for thin layer

Drying encompasses concurrent mass and energy transfer, detailing the impact of diverse process factors on moisture and shedding light on moisture removal mechanisms. The underlying mechanism's comprehension is intricate, and its exploration involves complexity [24]. Researchers have developed computer models to predict biomass moisture content based on temperature using thin layer drying principles [25]. These models offer insights into the physical processes of drying in a simpler and quicker manner compared to complex theoretical models. They have found application in studying agricultural product drying. Thin layer models assume uniform air characteristics throughout the layer due to the small product thickness, akin to drying a single layer of particles or slices fully exposed to air. These models can be classified into three categories:

• Theoretical Equations
• Semi-theoretical Equations
• Empirical equations [26].

Theoretical models are based on Fick's second law of diffusion, but they tend to be complex and less user-friendly. In contrast, semi-theoretical models adapt Fick's law along with Newton's law of cooling. Empirical models, drawn from experimental data and dimensional analysis, are simpler and more accessible.

### 1.4 Selection of drying models for the current study

The primary mechanism for moisture transfer to the surface is thought to be the diffusion process. Semi-theoretical models grounded in Fick's second law are easier to solve and require considerably less time than theoretical models. Additionally, they do not rely on assumptions regarding conductivity, mass diffusivity, or geometry, while still offering insights into the transfer process.

As was already indicated, the theoretical model (Fick's second law of diffusion) or its simplified version (Newton's law of cooling) is the primary source of semi-theoretical models. Here, two models from each category have been examined. To keep the work clearly comprehensible and to make the behavior predictions easier, only the most basic forms were utilized.

The Newton model, also referred to as the Lewis model, is the simplest due to its model constant and straightforward formulation, originating from Newton's law of cooling. The Page model serves as an empirical enhancement of the Newton model, significantly reducing errors by incorporating a new empirical dimensionless model constant (On the other hand, Fick's second law of diffusion's first term is represented by the Henderson and Pabis model. Again, a Logarithmic model is a modified form of Henderson and Pabis. These four models, namely Newton model, Page model, Logarithmic model and Henderson and Pabis model have therefore been selected for the current study.

## 2 Materials and methods

### 2.1 Algae production

The microorganism used in this study was *Chlorella vulgaris* (UTEX 2714). This strain of green algae was chosen for its relatively high lipid productivity, resilience to shear stress, significant CO2 absorption capacity, low nutrient requirements, rapid growth rate, and high photosynthetic efficiency [27]. Bristol medium^1^ was used for the algal growth. For production of *Chlorella vulgaris*, a 2L photo bioreactor was used. The bioreactor was obtained from the University of Texas, Austin. The photo-bioreactor is equipped with fully adjustable red (626 nm), green (525 nm), and blue (470 nm) LED lights, producing a total output of 43,200 mcd (MCD). It was operating on a cycle of 12 h on and 12 h off. After production, algae were harvested by sedimentation. The culture was kept without aeration for about 6 h, then culture media was removed, and the algae were collected from bottom. The algae were taken on piece of cloth and was hand-pressed to remove water from it.

### 2.2 Proximate analysis

Proximate analysis was performed on freshly harvested microalgae after a drying process of 3 h at 75 °C in a Memmert UN55 oven. Moisture content was determined according to ASTM D 95 by drying at 105 °C for 1 h. Volatile content was measured following ASTM D 5142 through ignition at 900 °C for 7 min. Ash content was determined by calcining at 700 °C for 20 min, also according to ASTM D 5142. Fixed carbon content was calculated by difference from the sum of moisture, volatile, and ash content.

### 2.3 Ultimate analysis

Elemental analysis for carbon, hydrogen, nitrogen, and sulfur was performed using a HEKAtech Euro EA-3000 analyzer. Oxygen content was determined by difference on an air-dry, ash-free basis. Calorific value was measured according to ASTM D 2015 using a bomb calorimeter. The calorimeter was pressurized with oxygen to approximately 25 bar before ignition of the sample with a copper wire. Calorific value was calculated based on the recorded temperature increase.

### 2.4 Thermal analysis

DSC-60 (Shimadzu, Japan) was used for the differential scanning calorimetry between room temperature and 600 °C. In a typical differential scanning calorimetry (DSC) experiment, the temperature of the sample unit, which consists of the sample and reference material, is increased at a rate of 10 K/min, while the temperature difference between the sample and the reference material is recorded as a function of temperature. DSC evaluates the sample's capacity to release or absorb energy, characterizing its endothermic and exothermic behavior throughout the drying process. The thermogravimetric experiments were conducted using a thermogravimetric analyzer (TGA; TGA-50; Shimadzu, Japan). In a typical run, approximately 5 mg algae sample was loaded in an aluminum cell. The sample was subsequently heated to 600 °C in a nitrogen flow of 10 mL/min at two different heating rates: 5 K/min and 20 K/min. The weight loss of the sample was monitored throughout the heating period. This data was used to calculate the derivate curve of weight loss, which is known as the differential thermogravimetry (DTG) curve. Data points for both techniques (TGA and DSC) were collected at a high sampling rate of 1 s intervals. This comprehensive dataset enables accurate analysis of thermal events and offers valuable insights into the material's behavior under different temperature conditions.

#### 2.4.1 Mathematical modelling of the drying curves

In existing literature, the drying kinetics of various biomass, particularly agricultural products, have often been investigated within isothermal conditions, leading to the proposal of several isothermal drying models. Numerous semi-theoretical models, as outlined in the introduction section, have been introduced. These models are typically obtained by simplifying the general series solution of Fick's second law and Newton's law of cooling, as previously discussed. For the analysis of *Chlorella vulgaris* drying kinetics, four selected models (outlined in Table 1) predominantly stem from Newton's law of cooling and Fick's second law. Other models are often variations of these fundamental models and due to their increased complexity, were not considered for this study.

**Table 1:** Four most popular isothermal drying kinetics model.

Serial No	Model Name	Model Equation
1	Newton/Lewis	MR = exp(-kt)
2	Page	MR = exp(-k $t^{n}$)
3	Henderson and Pabis	MR = a exp(-kt)
4	Logarithmic	MR = a exp(-kt) + c

The equations shown in Table, show the isothermal forms of the models. These equations were modified for the study of non-isothermal kinetics, by the introduction of a temperature dependent term. The procedure is described below.

The drying rate constant, denoted by the value k, is temperature dependent. Equation (1) shows how an Arrhenius-like relationship can explain this temperature dependence.

(1) $$k=k_{0}\;\exp\left(-\frac{E}{RT}\right)$$

where k_0_ represents the pre-exponential factor, E denotes the activation energy, R is the universal gas constant, and T indicates the absolute temperature.

As we have used non-isothermal heating, the temperature will increase with time. The time-temperature relationship for constant heating rate is shown in Equation (2).

(2) $$T=T_{0}+\beta t$$

where, T is the temperature at time t, T_0_ is the initial temperature and β is the heating rate.

After substituting these two relations in the isothermal drying kinetics model, non-isothermal drying kinetics models can be obtained. The non-isothermal expressions are shown in Equations. (3)–(6) and listed in Table 2. These derived non-isothermal drying kinetics models were used to determine the best suited drying kinetics model for *Chlorella vulgaris*.

**Table 2:** Derived non-isothermal model along with original form.

Model Name	Isothermal expression	Non-isothermal expression	
Newton/Lewis	MR = exp(-kt)	$\text{MR}=\exp\left[-k_{0}\;\exp\left(-\frac{E}{\text{RT}}\right)\;\frac{T-T_{0}}{\beta }\right]$	(3)
Page	MR = exp(-k $t^{n}$)	$\text{MR}=\exp\left[-k_{0}\;\exp\left(-\frac{E}{\text{RT}}\right){\left(\frac{T-T_{0}}{\beta }\right)}^{n}\;\right]$	(4)
Henderson and Pabis	MR = a exp(-kt)	$\text{MR}=a\;\exp\left[-k_{0}\;\exp\left(-\frac{E}{\text{RT}}\right)\;\frac{T-T_{0}}{\beta }\right]$	(5)
Logarithmic	MR = a exp(-kt) + c	$\text{MR}=a\;\exp\left[-k_{0}\;\exp\left(-\frac{E}{\text{RT}}\right)\;\frac{T-T_{0}}{\beta }\right]+c$	(6)

#### 2.4.2 Model fitting

Equation (7) was used to determine the moisture ratio based on the data acquired from TGA for drying.

(7) $$MR=\frac{m_{T}-m_{e}}{m_{i}-m_{e}}$$

Where, MR is the moisture ratio, m_T_ is the mass at temperature T, m_i_ is the mass at the initial temperature, while m_e_ is the mass at the conclusion of the drying process. The parameters of the non-isothermal kinetic models were obtained through non-linear regression of the experimental data. The quality of the fitted models can be assessed using various statistical metrics, including the correlation coefficient (R), coefficient of determination (R^2^), reduced chi-square (χ^2^), mean bias error (MBE), root mean square error (RMSE), sum of squares error (SSE), modeling efficiency (EF), mean percent error (MPE), and mean square error (MSE).

The quality of the fit was assessed using the value of R^2^ along with other criteria like χ^2^, RMSE, MBE, MPE, SSE, EF, and MSE. The coefficient of determination (R^2^) helps evaluate the linear relationship between experimental and model-calculated values and serves as a key criterion for selecting the best model. In this study, the parameters used to evaluate the fitness of the non-isothermal drying behavior have been shown in Equations (8), (9)).

(8) $$\chi^{2}=\frac{\sum_{i=1}^{N}{\left({MR}_{\exp,i}-{MR}_{pre,i}\right)}^{2}}{N-z}$$

and

(9) $$RMSE=\sqrt{\frac{1}{N}{\left({MR}_{exp,i}-{MR}_{pre,i}\right)}^{2}}$$

where, ${MR}_{\exp,i}$ is the experimental moisture ratio, N is the number of experimental data points and z is the number of parameters in the model.

## 3 Results and discussion

### 3.1 Proximate analysis

TGA and DSC analysis were done on freshly harvested algae paste. The moisture content of this algae paste was found to be 81.95 %. Table 3 depicts the proximate analysis of *Chlorella vulgaris*, considering both wet and dry bases, following 3 h of heating at 75 °C. The table reveals a substantial proportion of volatile content within the sample. These volatile constituents include a variety of hydrocarbons, acids, alcohols, esters, aldehydes, ketones, aromatics, and nitrogen compounds (such as pyrroles and amides), derived from the lipid, protein, and carbohydrate fractions of the microalgae. Additionally, the ash content is a notable parameter, encompassing the cumulative levels of iron, calcium, potassium, magnesium, sodium, and phosphorus present in *Chlorella*.

**Table 3:** Proximate analysis of *Chlorella vulgaris*.

Parameter	Wet basis	Dry basis
Moisture Content	6.67 %	–
Volatile	70.51 %	75.55 %
Ash Content	15.46 %	16.56 %
Fixed Carbon	7.36 %	7.89 %

### 3.2 Ultimate analysis

The results in Table 4 indicate the higher heating value of *Chlorella vulgaris*, along with elemental analysis detailing the composition percentages of C, H, N, S, and O.

**Table 4:** Ultimate analysis and heating value of *Chlorella vulgaris*.

Element	Mass percentage
C	43.02
H	8.53
N	14.40
S	3.5
O	30.55
Higher Heating Value (Dry Basis)	12.63 MJ/‌kg

### 3.3 Thermogravimetric Data analysis

**Fig. 1:**
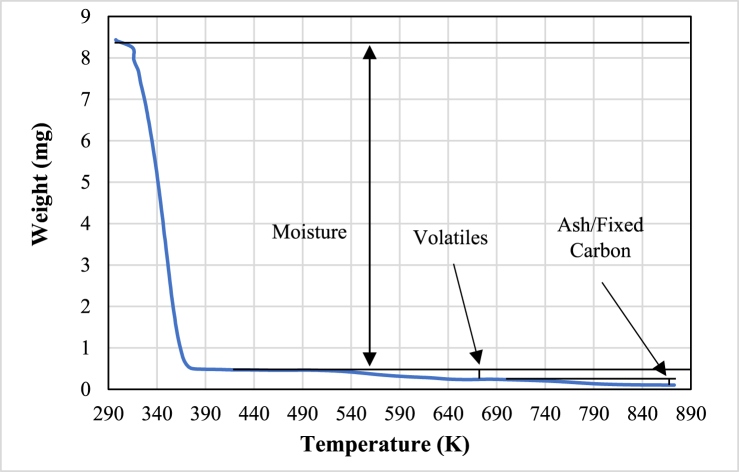
Thermogravimetric data.

The outcomes of the thermogravimetric experiments are displayed in Fig. 1, which shows the relation between temperature and weight loss. This weight loss is mainly due to moisture loss, volatile compounds loss and decomposition of the sample.

The initial weight of the wet sample was 8.435 mg. As the temperature gradually increased at a linear rate of 5 K/‌min, a reduction in weight became evident. During the initial mass loss stage, moisture content diminished rapidly with rising temperature. A distinct peak in mass loss emerged within the temperature range of 75 °C–80 °C, attributable to substantial moisture evaporation. At temperatures exceeding 150 °C, the sample underwent intricate thermal-chemical reactions, liberating diverse volatile substances and leading to a significant mass loss. These released components included hydrocarbons, acids, alcohols, esters, aldehydes, and ketones.

**Fig. 2:**
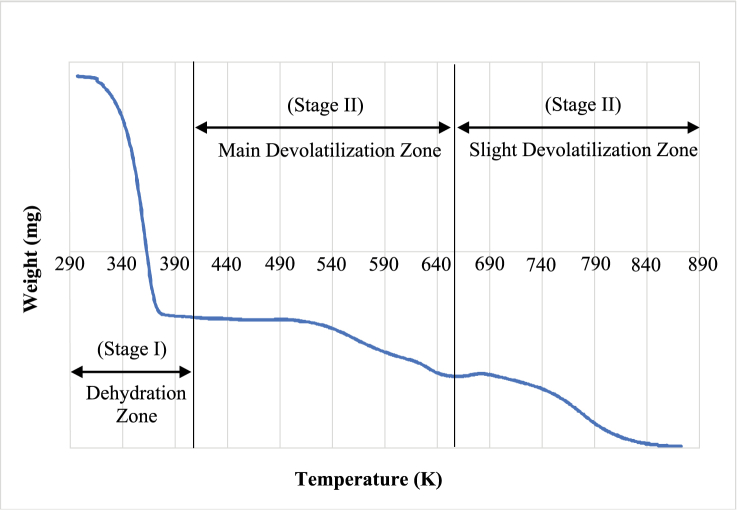
Thermogravimetric Data (log-log plot).

The log-log plot in Fig. 2 provides enhanced clarity regarding weight loss stages upon further heating. This graphical representation facilitates the distinct identification of different drying stages for *Chlorella vulgaris*. The biomass pyrolysis process was observed to comprise three stages: initial moisture evaporation (Stage I) from room temperature to 150 °C, significant devolatilization (Stage II) between 150 °C and 390 °C, and sustained minor devolatilization (Stage III) above 390 °C. This experimental analysis extended to 600 °C.

The weight difference between the beginning and the end of Stage I, encompassing up to 150 °C, represents the moisture content of the wet sample. Throughout devolatilization stages, weight loss indicates the cumulative volatile component, while the remaining weight at the experiment's conclusion signifies fixed carbon/‌ash content. Fig. 1 illustrates these parameters across diverse regions of the TGA curve. The data presented in Fig. 1 indicates an approximate 94 % moisture content in the sample. Notably, the central focus of this study pertains to Stage I, where moisture loss predominates.

**Fig. 3:**
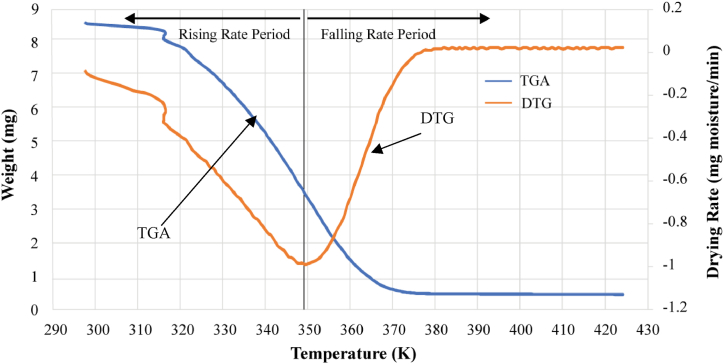
TGA and DTG showing peak point.

Both TGA and DTG data were integrated into Fig. 3, with the DTG curve pinpointing the peak of highest moisture removal rate at 75 °C. Subsequently, the rate diminishes and approaches near-zero levels around 150 °C or 423 K. The interval up to 150 °C primarily involves water/‌moisture loss. The TGA/DTG curves level out as the temperature gets closer to 150 °C, signifying the start of the organic components in the biomass's thermal breakdown. This temperature, 150 °C, generally signifies the end of dehydration.

From Fig. 1, Fig. 2, Fig. 3 the absence of a constant rate period is evident, and noticeable peaks in water loss emerge around 75 °C. Utilizing these peaks as reference points, As illustrated in Fig. 3, the moisture evaporation phase can be divided into two periods: a rising rate period and a falling rate period.

Water-biomass bonding is strongly correlated with changes in the rate of water loss. Samples that are moist contain two different types of moisture: bound water that has adsorbed within pores and free water that sticks to the surface. Weakly bonded free water evaporates before 90 °C. On the other hand, over 120 °C, bound water—which is held together by strong bonds and is situated deeper—completely separates. Free water rapidly diffuses outward to meet gasification requirements during the rising rate period, increasing the drying rate as significant amounts of steam are emitted. Thus, this stage corresponds to the evaporation of free water from algae before to the peak of water loss.

#### 3.3.1 Effects of heating rate on drying curves

In this study, two heating rates (20 K/min and 5 K/min) were employed. The mass loss curve moved to the left with an increase in heating rate, indicating a quicker decrease in the moisture ratio.

**Fig. 4:**
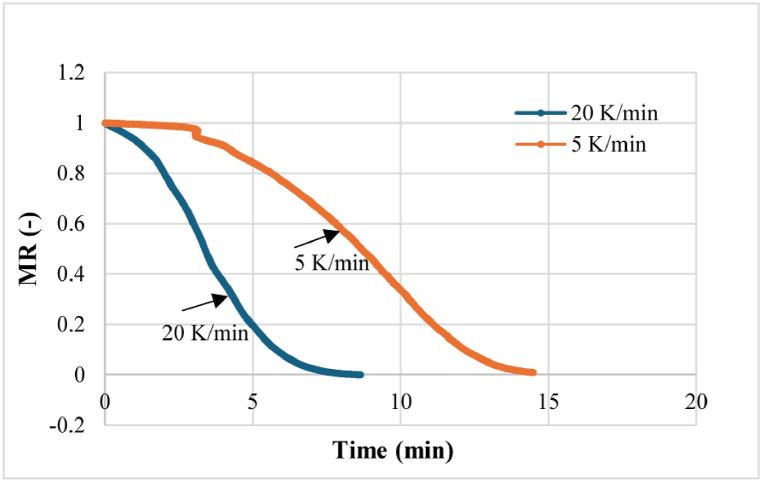
Effects of heating rate on moisture removal.

The shortest time to reach the final moisture content was noted at the heating rate of 20 K/min. Each moisture ratio loss curve primarily exhibited two distinct regions. In Fig. 4, The initial region is almost close to the horizontal line (highly noticeable in 5 K/min rate curve) is the heating stage. This stage lasted for approximately 3 min. This was followed by a sharp fall in moisture ratio loss. It was the main drying region. It took 8.5–14.5 min to end the drying process. The time required in drying at rate 5 K/min is much higher than 20 K/min. As the drying rate is slower the time would be higher, this is obvious. But, at 5 K/‌min drying rate the moisture removal stopped at around 100 °C, whereas it rose up to 200 °C for 20 K/min drying rate. At higher heating rates, there is not enough time for the moisture to migrate from the sample. However, at lower heating rates, there is more time for the moisture to migrate from the sample.

### 3.4 Model fitting

Four models were used to fit the data obtained from TGA. Three parameters were considered as the criteria to determine the best fit as mentioned earlier. These are R^2^, χ2 and RMSE. R^2^ value close to 1 means better fitting. The closer to 1 the better the fit. On the other hand, for χ2 and, the less the value the better the fit.

#### 3.4.1 Fitting of 5 K/min drying rate data

**Fig. 5:**
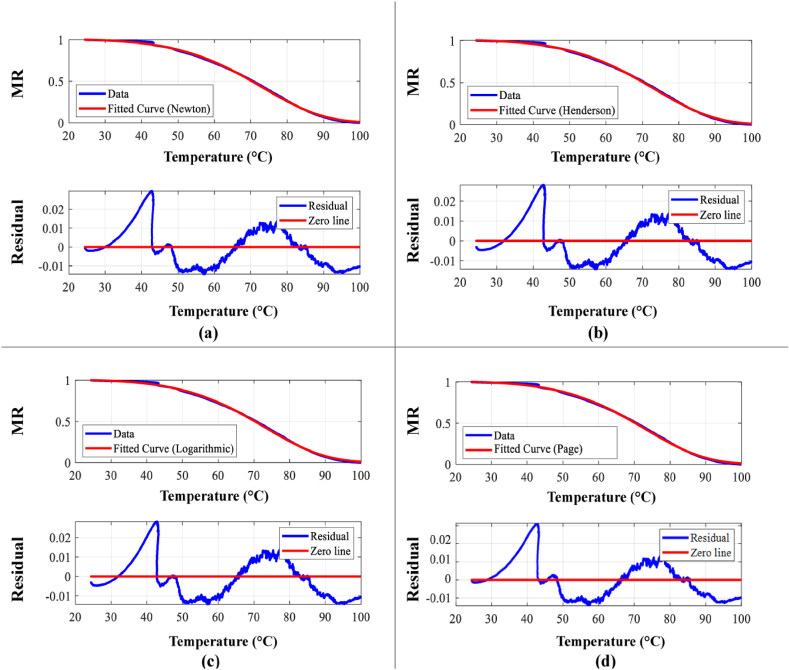
Model fit for (a) Newton model, (b) Henderson and Pabis, (c) Logarithmic model, (d) Page model.

Fig. 5(a–d) presents a comparative analysis of experimental and modeled moisture ratio (MR) data against temperature (T) for each of the four drying models. The corresponding residual errors between experimental and predicted MR values are displayed below each plot, providing insights into model performance.

The list of model parameters along with the goodness of fitting values obtained from all four drying kinetics models have been shown in Table 5.

**Table 5:** Data showing the quality of fit using all four models.

Models	R^2^	χ^2^	RMSE	a (−)	c (−)	E (kJ.mol^−1^)	k (min^−1^)	n (−)
Newton	0.99951	1.117E-04	0.0106	–	–	49.87	2.89E6	–
Henderson and Pabis	0.99940	1.110E-04	0.0152	1.002	–	49.46	2.52E5	–
Logarithmic	0.99960	1.111E-04	0. 0105	1.003	2.33E-6	49.46	2.52E6	–
Page	0.99968	1.107E-04	0.0105	–	–	52.71	1.03E7	0.873

From the data shown in Tables 5 and it can be seen that all four models show good fitting which supports results obtained from literature for different kinds of agricultural products [24,[28], [29], [30]]. Among the four, the Page model shows the best fitting. **R**^**2**^ value is more closed to 1 than other three models, while having lowest value of **χ2** value. In terms of **RMSE** both Logarithmic and Page model shows equal value. So, among the four models, both Page and Logarithmic model shows the better match while Page is the perfect one.

#### 3.4.2 Fitting of 20 K/min drying rate data

**Fig. 6:**
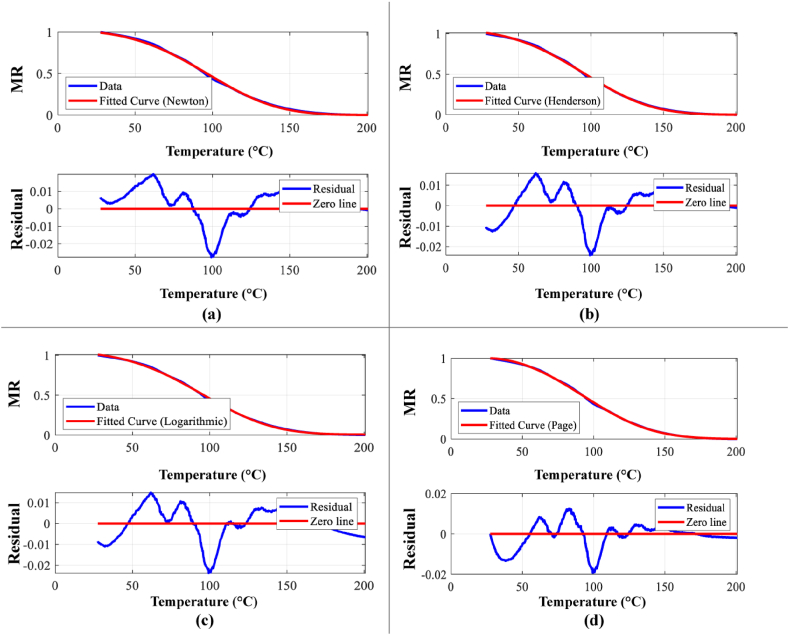
Model fit for (a) Newton model, (b) Henderson and Pabis, (c) Logarithmic model, (d) Page model.

Fig. 6(a–d) presents a comparative analysis of experimental and modeled moisture ratio (MR) data against temperature (T) for each of the four drying models. The corresponding residual errors between experimental and predicted MR values are displayed below each plot, providing insights into model performance. The list of model parameters along with the goodness of fitting values obtained from all four drying kinetics models are shown in Table 6.

**Table 6:** Data showing the quality of fit using all four models along with calculated parameters.

Models	R^2^	χ^2^	RMSE	a	c	E (kJ.mol^−1^)	k (min^−1^)	n
Newton	0.99939	7.588E-05	0.00869	–	–	19.62	119.58	–
Henderson and Pabis	0.99947	6.616E-05	0.00811	1.01	–	18.98	99.02	–
Logarithmic	0.99951	6.097E-05	0.00778	1.003	5.72E-3	19.48	117.1	–
Page	0.99969	3.842E-05	0.00618	–	–	10.24	2.83	1.58

Similarly, from the Table 6 all four models show good fitting. Among the four, Page model shows the best fitting. R^2^ value was more closed to 1 than other three models, while having lowest value of χ2 and RMSE value. Among the four models, both Page and Logarithmic model show the better match while Page is the best.

It can be concluded that in both cases, the Page model fits the drying behavior very well. Data obtained from both drying rate (5 K/‌min and 20 K/min) can easily be predicted by this model.

### 3.5 Fitting of drying curve for the parameters derived from another drying rate

**Fig. 7:**
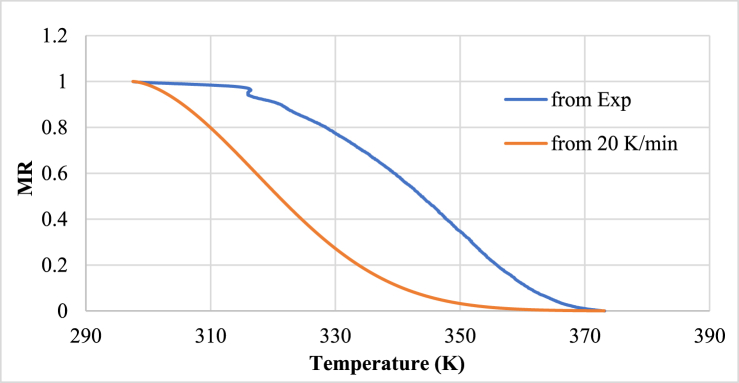
Drying curve of heating rate 5 K/min with parameters obtained from heating rate 20 K/min.

Two different drying rates have been used in this study. Under this section, Page equation with parameters obtained from one heating rate has been compared with the experimental value obtained at other heating rate. First, the drying curve obtained at a heating rate of 5 K/min from the experiment has been compared with values obtained from using parameters value obtained from drying curve of heating rate 20 K/min using the Page model. The comparison has been shown in Fig. 7.

The fittings are not good at all. So, the parameters obtained from 20 K/min heating rate have not succeeded to predict the drying behavior at another heating rate.

The next plot as shown in, shows the opposite combination. The drying curve of heating rate 20 K/‌min fitted with parameters value obtained from drying curve of heating rate of 5 K/min using the Page model.

**Fig. 8:**
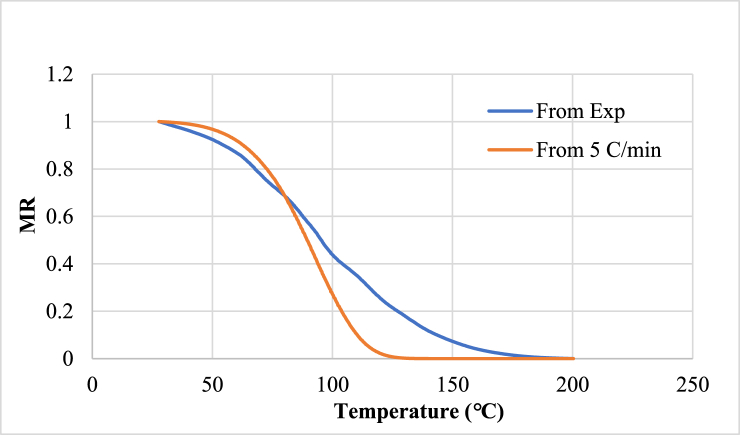
Drying curve of heating rate 20 K/min with parameters obtained from heating rate 5 K/min.

From Fig. 8, it can be seen that the parameters derived from one drying curve with a drying rate fails to fit the other one. This happens because drying rate has an effect on drying kinetics.

In this study, to measure the drying kinetics of *Chlorella vulgaris* using TGA analysis, two different fixed heating rates: 5 °C/min and 20 °C/min have been used. Interestingly, the best-fit model for one heating rate failed to predict the experimental values for the other heating rate. This finding underscores the complexity of the drying process and emphasizes the necessity of selecting suitable models for varying conditions.

**Fig. 9:**
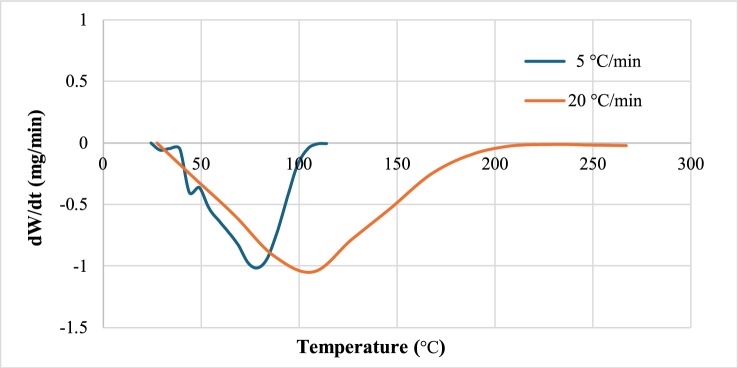
Effects of heating rate on weight loss derivative.

The activation energies decreased as the heating rate increased. This is because a higher heating rate accelerates the drying process into a higher temperature range, resulting in an increase in temperature within the biomass. The weight loss derivative reached its highest value of 1.047 mg/min at the heating rate of 20 K/min at about 107 °C as seen on Fig. 9. Due to these factors, parameters obtained from one drying rate do not align with the drying curve of another drying rate.

At heating rates of 20 K/min and 5 K/min, the activation energies (E) obtained were 10.24 kJ/mol and 52.71 kJ/mol, respectively. This stands for the energy barrier that must be overcome to dry microalgae. The literature states that green microalgae (Chlorella sp.) have an activation energy of 28.7 kJ/mol [31]. The convective drying of a group of microalgae has been observed to have an activation energy of approximately 31.83 kJ/mol, while *Macrocystis pyrifera*, a type of brown algae, has an activation energy of 19.87 kJ/mol [32,33]. It has been found that the activation energy required to dry the majority of agricultural products ranges from 18 to 49.5 kJ/mol [34]. Tomato drying was shown to have an activation energy ranging from 59.6 to 70.2 kJ/mol, according to Doymaz and Özdemir (2014). The value found in this study is within the ranges that other investigations have reported.

The broader applicability of these results extends to various contexts, particularly in supporting the microalgae drying process for biofuel and nutraceutical production. Understanding the drying kinetics at different heating rates provides valuable insights into optimizing large-scale drying processes to maximize efficiency and product quality. For biofuel production, where rapid and efficient drying is critical to maintain energy yield and minimize processing costs, these findings can guide the selection of optimal drying conditions. By tailoring the drying process to the specific thermal properties of microalgae, it is possible to achieve consistent moisture removal and prevent thermal degradation of valuable compounds.

In the context of nutraceutical production, where preserving bioactive compounds, pigments, and nutrients is essential, the ability to accurately model and predict drying behavior is crucial. The insights gained from TGA analysis at different heating rates can inform the development of drying protocols that maintain the integrity of these sensitive compounds. For instance, slower heating rates might be preferred to avoid thermal degradation, even though they might not be as efficient in terms of time and energy consumption.

Moreover, the variability in model performance across different heating rates underscores the need for flexibility and adaptability in industrial drying processes. It suggests that a one-size-fits-all approach may not be suitable for all microalgae species or products. Instead, developing a range of models that can be applied based on specific drying conditions and product requirements can enhance the reliability and effectiveness of the drying process.

In summary, the failure of a single model to predict drying kinetics across different heating rates demonstrates the necessity for diverse modeling approaches tailored to specific drying conditions. These results are broadly applicable in optimizing microalgae drying processes for biofuel and nutraceutical production, ensuring efficiency, and preserving product quality. Additionally, the activation energy and drying rate constant values for *Chlorella vulgaris* found in this study align with those documented in the literature for other microalgal species. This indicates that the drying behavior of *Chlorella vulgaris* follows similar kinetics, providing a reliable basis for scaling up the drying process for biofuel and nutraceutical production.

### 3.6 DSC analysis for drying

**Fig. 10:**
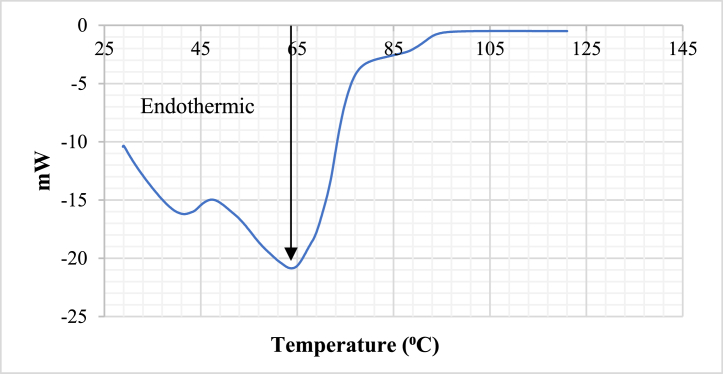
DSC curve.

Fig. 10 shows the heat requirement during the drying process. The DSC curve shows two distinct peaks. The first peak is at around 42 °C and the second one is at around 64 °C. The first peak is mainly due to free water present in the sample.

While after 42 °C, not only free water but also bound water contribute to the heat requirement. Apart from these there is a shoulder at around 89 °C.

**Fig. 11:**
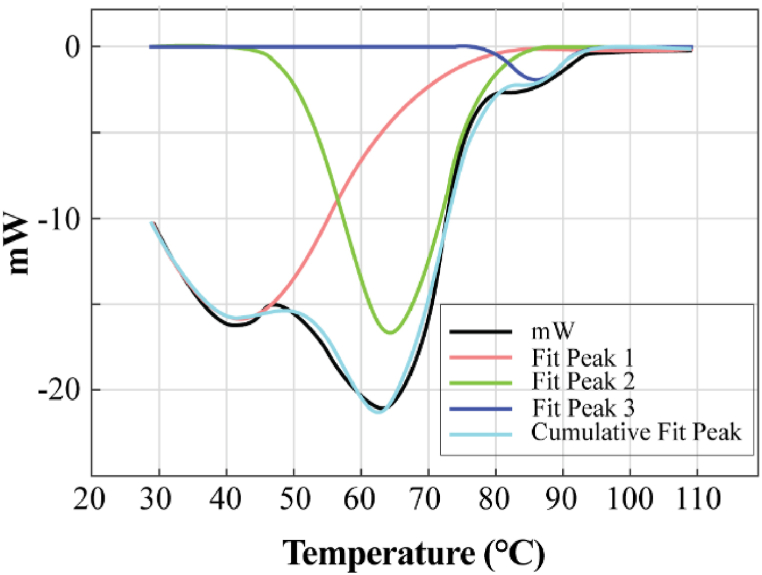
DSC curve presented by three gaussian curve.

Each peak in the DSC curve indicates a phase, in this case different phases of water. Therefore, the DSC curve indicated the presence of two or three types of moisture being present in the sample. The DSC curve is therefore fitted with three separate gaussian curves as shown on Fig. 11.

**Fig. 12:**
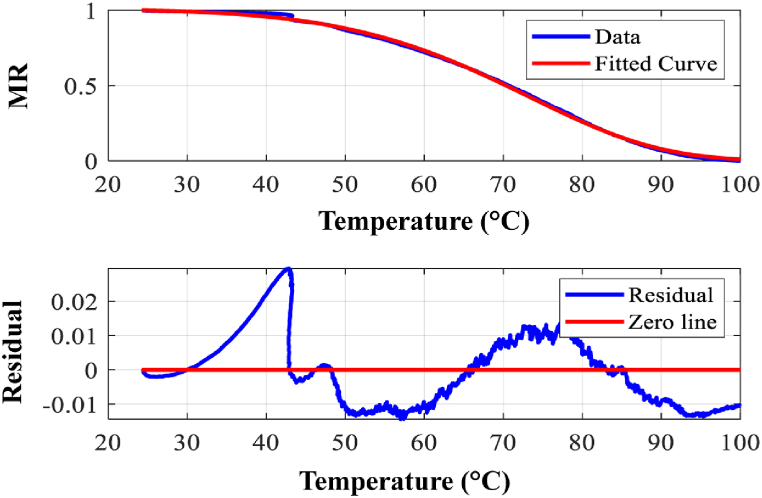
Curve fit for three curves fit.

Observably, the three curves have effectively recreated the experimental DSC curve, suggesting the existence of three distinct moisture types. To delve deeper into the moisture categories, TGA data analysis can be conducted. To depict these diverse moisture types, three Gaussian curves were chosen, with the intention of utilizing Newton's model for this endeavor. The fittings of calculated data with experimental data have been shown in Fig. 12.

From the Figure the matching is quite good. The results of the parameters along with their contribution has been shown on Table 7.

**Table 7:** Data for three curve fit.

Moisture Type/Region	E	K	%	R^2^ value
Curve 1	45.54	6.628429 E05	0.9892	0.9971
Curve 2	13.79	1.475588e E06	0.0001	
Curve 3	25.09	1.964289 E03	0.0107	

**Fig. 13:**
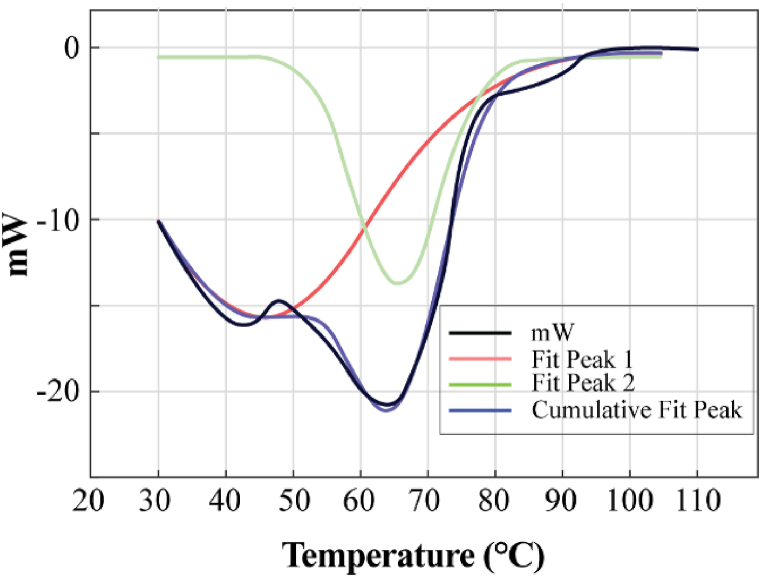
DSC curve presented by two gaussian curve.

The R square value of the fit is 0.9971. From the Tables 7 and it is apparent that the contribution of all three curves is not the same. The results show the maximum contribution is coming from curve 1. Another fit was plotted considering only two regions. The result has been shown as follows in Fig. 13.

**Fig. 14:**
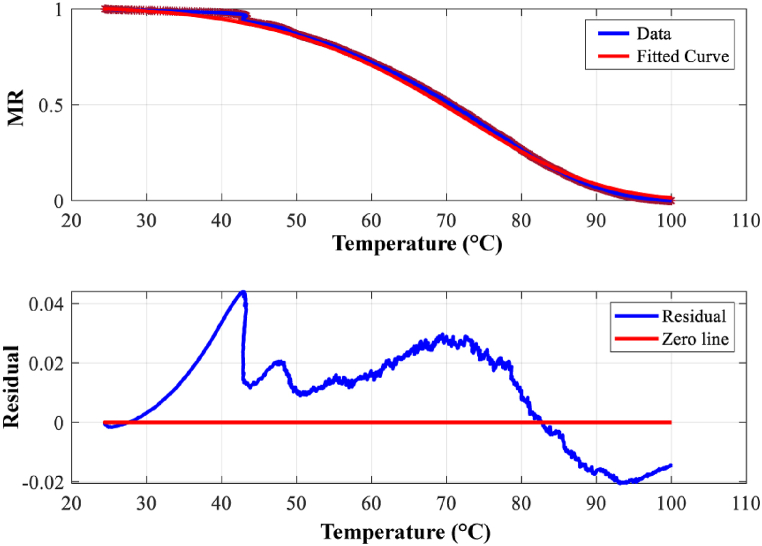
Curve fit for two curve fit.

Likewise, it is evident that the two curves have effectively mirrored the experimental DSC curve in this scenario as well. This observation suggests the potential existence of two distinct moisture types. To delve deeper into the moisture categorization, TGA data analysis can be pursued. To represent these two diverse moisture types, two equations were chosen, aligning with the utilization of Newton's model for this purpose as well. The alignment between calculated data and experimental data is illustrated in Fig. 14.

Also, the fact was verified with two equations fit and the plot data is presented in Table 8.

**Table 8:** Data for two curve fit.

Moisture Type/Region	E	k	%	R^2^
Curve 1	49.87	2.8929E05	0.999	0.9939
Curve 2	3.56E3	3.6106E06	0.001	9939

The fact observed in the earlier three equation plot is also present here. Here the fitting is good, but the first curve consists of almost 99.9 % of the total drying curve. So, it can be concluded that there present three different kinds of moisture. But, for drying of microalgae only single type of moisture (Free water) removal is the key fact.

## 4 Conclusion

In conclusion, the non-isothermal drying kinetics of *Chlorella vulgaris* species were rigorously investigated using TGA with heating rates of 5 K/min and 20 K/min. The choice of this specific algae species was driven by its promising attributes, including high lipid content, ease of cultivation, and potential for large-scale oil production. Through proximate and ultimate analyses, we unveiled significant volatile content (>70 %) and an ash content of around 15 % on a wet basis, along with a noteworthy heating value of approximately 12.63 MJ/kg. The TGA and DTG data illuminated the optimal drying temperature, suggesting that the range of 75 °C–80 °C is most suitable for biofuel production. Notably, Page's model emerged as the most fitting among the kinetic models tested for both heating rates, with activation energy values of 49–53 kJ/mol and 18–20 kJ/mol for 5 K/min and 20 K/min heating rates, respectively. The presence of distinct moisture types was observed from the DSC curve, but the drying process primarily involves the removal of a single type of moisture (free water), making single equation fitting appropriate. These findings provide important insights into the drying behavior of *Chlorella vulgaris*, paving the way for further investigations into its applications in biofuel production and nutraceutical value assessment at varying drying temperatures.

## Data availability statement

Data will be made available on request.

## CRediT authorship contribution statement

**Debabrata Karmakar:** Software, Resources, Methodology, Formal analysis, Data curation. **Nishat Tasnim:** Writing – review & editing. **Md. Rakibul Hasan:** Supervision. **Md. Saddam Hossain:** Resources. **Paroma Arefin:** Formal analysis, Data curation. **Dip Bhowmik:** Resources, Project administration. **Yead Morshed Nibir:** Resources, Methodology, Investigation. **Kazi Bayezid Kabir:** Supervision, Project administration, Methodology, Investigation, Conceptualization. **Rezaul Karim:** Supervision.

## Declaration of competing interest

The authors declare that they have no known competing financial interests or personal relationships that could have appeared to influence the work reported in this paper.

## Footnotes

1 http://web.biosci.utexas.edu/utex/Media%20PDF/bristol-medium.pdf.
